# Large-scale comparative review and assessment of computational methods for phage virion proteins identification

**DOI:** 10.17179/excli2021-4411

**Published:** 2022-01-03

**Authors:** Muhammad Kabir, Chanin Nantasenamat, Sakawrat Kanthawong, Phasit Charoenkwan, Watshara Shoombuatong

**Affiliations:** 1 School of Systems and Technology, Department of Computer Science, University of Management and Technology, Lahore, Pakistan, 54770; 2 Center of Data Mining and Biomedical Informatics, Faculty of Medical Technology, Mahidol University, Bangkok, Thailand, 10700; 3 Department of Microbiology, Faculty of Medicine, Khon Kaen University, Khon Kaen, Thailand, 40002; 4 Modern Management and Information Technology, College of Arts, Media and Technology, Chiang Mai University, Chiang Mai, Thailand, 50200

**Keywords:** phage virion protein, bioinformatics, classification, machine learning, feature representation, feature select

## Abstract

Phage virion proteins (PVPs) are effective at recognizing and binding to host cell receptors while having no deleterious effects on human or animal cells. Understanding their functional mechanisms is regarded as a critical goal that will aid in rational antibacterial drug discovery and development. Although high-throughput experimental methods for identifying PVPs are considered the gold standard for exploring crucial PVP features, these procedures are frequently time-consuming and labor-intensive. Thusfar, more than ten sequence-based predictors have been established for the *in silico* identification of PVPs in conjunction with traditional experimental approaches. As a result, a revised and more thorough assessment is extremely desirable. With this purpose in mind, we first conduct a thorough survey and evaluation of a vast array of 13 state-of-the-art PVP predictors. Among these PVP predictors, they can be classified into three groups according to the types of machine learning (ML) algorithms employed (i.e. traditional ML-based methods, ensemble-based methods and deep learning-based methods). Subsequently, we explored which factors are important for building more accurate and stable predictors and this included training/independent datasets, feature encoding algorithms, feature selection methods, core algorithms, performance evaluation metrics/strategies and web servers. Finally, we provide insights and future perspectives for the design and development of new and more effective computational approaches for the detection and characterization of PVPs.

## Introduction

Bacteriophages are viruses that may infect bacteria and replicates within them. They are obligate intracellular parasites that are widely distributed in areas populated by bacterial hosts, such as soil, water, and animal or human intestines, with a viral population estimated to be higher than 10^31^ particles (Clark and March, 2006[19]; Lekunberri et al., 2017[38]; Lyon, 2017[41]). Phage virions are made up of genetic material (DNA or RNA) and a coat of structural proteins (or virion proteins) that can interact with host cell receptors and insert their genome into the cell via one of two basic strategies: the lytic or lysogenic cycle (Roach and Donovan, 2015[49]). Lytic phages harness the host cell's biological machinery to synthesis their DNA and the remaining proteins needed to produce new phage particles. The new genomes are then packed into the head and phage progeny construct. Finally, phages lyse host cells and release additional phage particles (about 100-200 offspring) into the environment (Doss et al., 2017[23]; Roach and Donovan, 2015[49]). Prophages are lysogenic phages (temperate) that integrate their genome into the bacterial chromosome and become part of the host without killing the cell. The prophage genome is reproduced with the host chromosome and passed on to new daughter cells in a dependent manner. Under severe conditions, the viral genome can be extracted from the chromosome of the host bacterium, and lysogenic phages can go through a lytic cycle to produce new particles (Samson et al., 2013[52]).

Protein arrays and mass spectrometry are two prominent examples of well-known experimental approaches used to discover and characterize PVPs (Lavigne et al., 2009[36]; Yuan and Gao, 2016[63]). As these techniques are time-consuming, labor-intensive and costly nature. As a result, the development of computational models capable of swiftly and accurately identifying PVPs is critical. Currently, there are 13 state-of-the-art predictors that are based on a wide range of machine learning (ML) techniques (Arif et al., 2020[2]; Charoenkwan et al., 2020[11]; Charoenkwan et al., 2020[14]; Ding et al., 2014[22]; Fang and Zhou, 2021[25]; Feng et al., 2013[27]; Han et al., 2021[28]; Manavalan et al., 2018[42]; Pan et al., 2018[48]; Ru et al., 2019[51]; Seguritan et al., 2012[53]; Tang et al., 2016[56]) for PVPs identification while two review papers (Meng et al., 2020[43]; Nami et al., 2021[44]) have emerged in this aspect. These review articles provided a good summary on the current state-of-the-art of PVPs identification. In spite of their merit, the overall scope of these articles is quite outdated. These review articles provided limited coverage on important aspects that are beneficial for the development of more accurate PVP predictors including training/independent datasets, core algorithms and webserver.

Herein, we propose the following important issues that needs to be addressed. Firstly, a more updated and comprehensive review is highly needed. These review papers did not provide a comprehensive survey on all of the currently available PVP predictors. A comprehensive review of all existing methods will be very useful for experimental scientists in selecting suitable PVP predictors for identifying investigated unknown sequences. Secondly, exploration on the underpinnings contributing to the development of more accurate PVP predictors would also be immensely useful.

Motivated by these aforementioned considerations, we herein conduct the first comprehensive overview and assessment of a large collection of 13 state-of-the-art PVP predictors. Table 1(Tab. 1) (References in Table 1: Arif et al., 2020[2]; Charoenkwan et al., 2020[11][14]; Ding et al., 2014[22]; Fang and Zhou, 2021[25]; Feng et al., 2013[27]; Han et al., 2021[28]; Manavalan et al., 2018[42]; Pan et al., 2018[48]; Ru et al., 2019[51]; Seguritan et al., 2012[53]; Zhang et al., 2015[64]) summarizes these sequence-based PVP predictors along with their employed feature encoding algorithms, feature selection methods, ML algorithms and performance evaluation metrics/strategies. In particular, we reviewed all datasets used in the development of current PVP predictors. Details on these datasets are summarized in Table 2(Tab. 2) (References in Table 2: Arif et al., 2020[2]; Charoenkwan et al., 2020[11][14]; Ding et al., 2014[22]; Fang and Zhou, 2021[25]; Feng et al., 2013[27]; Han et al., 2021[28]; Manavalan et al., 2018[42]; Pan et al., 2018[48]; Ru et al., 2019[51]; Seguritan et al., 2012[53]; Tan et al., 2018[55]; Zhang et al., 2015[64]). Furthermore, we had also examined training/independent datasets, feature encoding algorithms, feature selection methods and ML algorithms. From amongst the current predictors, they can be categorized into three groups including conventional machine learning-based methods (i.e. iVIREONS (Seguritan et al., 2012[53]), Feng et al.'s method (Feng et al., 2013[27]), PVPred (Ding et al., 2014[22]), PVP-SVM (Manavalan et al., 2018[42]), PhagePred (Pan et al., 2018[48]), Tan et al.'s method (Tan et al., 2018[55]), Ru et al.'s method (Ru et al., 2019[51]), Pred-BVP-Unb (Arif et al., 2020[2]) and PVPred-SCM (Charoenkwan et al., 2020[11])), ensemble-based methods (i.e. Zhang et al.'s method (Zhang et al., 2015[65]), Meta-iPVP (Charoenkwan et al., 2020[11]) and iPVP-MCV (Han et al., 2021[28])) and deep learning-based methods (i.e. VirionFinder (Fang and Zhou, 2021[25])). Subsequently, we performed a comparative result analysis on three well- known benchmark datasets.

Finally, we summarize some key insights and future perspectives for the design and development of new next-generation computational methods for PVPs identification and characterization.

## Materials and Methods

### Benchmark datasets

In the viewpoint of ML, the construction of a high-quality dataset is one of the quintessential step in the development of reliable computational predictors. The following steps were conducted in the establishment of a high-quality PVP dataset. In the first step, all sequences were experimentally verified as PVPs and non-PVPs. In the second step, PVPs and non-PVPs containing non-standard letters (e.g. “B”, “X” or “Z”) were excluded. In the final step, the sequence identity threshold was set to be in the range of 0.3-0.4 in order to avoid sequence redundancy. Details on different datasets used for constructing currently available PVP predictors are summarized in Table 2(Tab. 2). From amongst these datasets, there are three popular benchmark datasets consisting of *Feng2013* dataset (Feng et al., 2013[27]), *Manavalan2018* (Manavalan et al., 2018[42]) and *Charoenkwan2020_2.0* (Charoenkwan et al., 2020[14]), which are frequently used for the development of existing PVP predictors consisting of PVPred (Ding et al., 2014[22]), Zhang et al.'s method (Zhang et al., 2015[64]), PVP-SVM (Manavalan et al., 2018[42]), PhagePred (Pan et al., 2018[48]), Tan et al.'s method (Pan et al., 2018[48]), Pred-BVP-Unb (Arif et al., 2020[2]), PVPred-SCM (Charoenkwan et al., 2020[11]), Meta-iPVP (Charoenkwan et al., 2020[14]) and iPVP-MCV (Han et al., 2021[28]). In 2012, the *Seguritan2012* dataset (Seguritan et al., 2012[53]) was released as the first dataset that has been used for the development of a sequence-based predictor for *in silico* PVP identification that consisted of 6303 PVPs and 6303 non-PVPs. Afterwards, the *Feng2013 *dataset was introduced by Feng et al. (2013[27]) and it represents the first high-quality dataset to apply a CD-HIT threshold of 0.4 that eventually led to a dataset of 99 PVPs and 208 non-PVPs. Particularly, the Feng2013 dataset can be downloaded from http://lin-group.cn/server/PVPred. In 2018, Manavalan et al. constructed the *Manavalan2018 *dataset (Manavalan et al., 2018[42]) by combining the *Feng2013* dataset with a new independent dataset containing 30 PVPs and 64 non-PVPs, which were manually collected from several PVP studies (Feng et al., 2013[27]; Pan et al., 2018[48]; Zhang et al., 2015[64]). The *Manavalan2018* dataset can be downloaded from http://www.thegleelab.org/PVP-SVM/PVP-SVM.html. Recently, our group constructed an up-to-date dataset consisting of 313 PVPs and 957 non-PVPs, which were downloaded from the UniProt database (release 2019_11) (Charoenkwan et al., 2020[14]). To solve the overestimation issue that typically occurs during model optimization, the set of 313 PVPs and 957 non-PVPs were randomly divided into training and independent datasets using the 80/20 split ratio. This led to a training dataset consisting of 250 PVPs and 250 non-PVPs while the independent datasets consisted of 63 PVPs and 63 non-PVPs. These training and independent datasets are referred as the *Charoenkwan2020_2.0* dataset in this review. The Charoenkwan2020*_2.0* dataset can be downloaded from https://github.com/Shoombuatong/Dataset-Code/tree/master/PVP.

Several observations can be made from Table 1(Tab. 1). Firstly, the *Feng2013* dataset (Feng et al., 2013[27]) was most frequently used for developing PVP predictors and for assessing their cross-validation performance (i.e. Feng et al.'s method (Feng et al., 2013[27]), PVPred (Ding et al., 2014[22]), PVP-SVM (Manavalan et al., 2018[42]), PhagePred (Pan et al., 2018[48]), Tan et al.'s method (Tan et al., 2018[55]), Pred-BVP-Unb (Arif et al., 2020[2]), PVPred-SCM (Charoenkwan et al., 2020[11]) and iPVP-MCV (Han et al., 2021[28])). Secondly, the independent dataset derived from the *Manavalan2018* dataset (Manavalan et al., 2018[42]) was the most frequently used one for assessing the independent test results of variant PVP predictors consisting of PVP-SVM (Manavalan et al., 2018[42]), Tan et al.'s method (Tan et al., 2018[55]), Pred-BVP-Unb (Arif et al., 2020[2]), PVPred-SCM (Charoenkwan et al., 2020[11]) and iPVP-MCV (Han et al., 2021[28]). Thirdly, the *Charoenkwan2020_2.0* dataset (Charoenkwan et al., 2020[14]) provided the largest number of PVPs and non-PVPs.

### Feature encoding schemes

Machine-learning based PVP predictors require the extraction of feature information from the sequence. PVPs have been encoded into fix-length feature vectors using a variety of features encoding approaches (Arif et al., 2020[2]; Charoenkwan et al., 2020[11]; Ding et al., 2014[22]; Feng et al., 2013[27]; Han et al., 2021[28]; Manavalan et al., 2018[42]; Pan et al., 2018[48]; Ru et al., 2019[51]; Zhang et al., 2015[64]). In the present PVP predictors, there are five major types of feature descriptors, as shown in Table 3(Tab. 3) (References in Table 3: Arif et al., 2020[2]; Charoenkwan et al., 2020[11]; Ding et al., 2014[22]; Feng et al., 2013[27]; Han et al., 2021[28]; Manavalan et al., 2018[42]; Pan et al., 2018[48]; Ru et al., 2019[51]; Seguritan et al., 2012[53]; Zhang et al., 2015[64]). Composition features (AAC, AKSNG, DPC, GGAP, and SAAC), position features (bi-PSSM, bi-Profile Bayes, DP-PSSM, PSSM, PSSM-AAC, PSSM-Composition, and PSSM Profiles), physicochemical properties (AACPCP, CTD, PAAC, and PCP), meta-based features (i.e. PFs), and structure features (i.e. PFs) were (i.e. Seq-Str). The most widely used descriptors are AAC, CTD, DPC, GGAP, and PSSM, as shown in Table 2(Tab. 2) and their definitions are given below. 

AAC descriptors represent the occurrence frequency of standard amino acids in a protein sequence (Charoenkwan et al., 2021[12], 2020[13], 2020[14]). The percentage composition (*aa*(*i*)) of the *i^th^* amino acid is represented by:


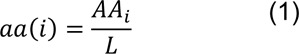


where *AA_i_* is the count or occurrence for the *i^th^* amino acid and *L* is the length of the protein. DPC descriptors represent the occurrence frequency of all possible dipeptides in a protein sequence (Charoenkwan et al., 2021[9], 2020[11], 2013[15], 2020[16], 2020[17]). The percentage composition (*dp*(*i*)) of the *i^th^* dipeptide is represented by:


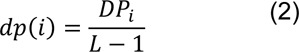


Where *DP_i_* is the count of occurrences of the *i^th^* dipeptide. Final vectors for AAC and DPC descriptors are represented as 20- and 400-dimension (20-D and 400-D, respectively) feature vectors, respectively. The GGAP descriptor is another variation of the DPC descriptor (*g* = 0) by representing the occurrence frequency of any two interval amino acids (*aa*_i_, *aa*_j_; *j* - *i *> 1) in a given protein **P** (Ding et al., 2014[22]; Pan et al., 2018[48]). This descriptor can be formulated as follows:


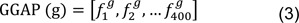


where *f_i_^g^* is the percentage composition of the *i^th^* (*i* = 1,2,…,400) *g-*gap dipeptide.


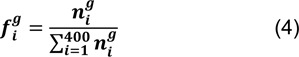


where *n_i_^g^* represents the percentage composition of *i^th^* *g-*gap dipeptide in a given protein **P**. The final vector for GGAP is a 400-D feature vector.

The CTD descriptor describes the amino acid characteristics of protein sequences in general (Li et al., 2006[40]). Combination (C), transformation (T), and distribution (D) are three separate feature descriptors provided by this method (Dubchak et al., 1995[24]). Hydrophobicity, normalized van der Waals volume, polarity, polarization, charge, secondary structure, and solvent accessibility are among the 13 physicochemical properties used to create these three separate feature descriptors (Chen et al., 2018[18]). Particularly, CTDC, CTDD and CTDT represent 39-D, 195-D and 39-D feature vectors, respectively (Arif et al., 2020[2]; Zhang et al., 2015[64]). Further details on CTDC, CTDD and CTDT descriptors are described in the work by Li et al. (2006[40]).

The PSSM descriptor can be extracted by the evolutionary profile feature representation method. This descriptor is a position-based feature encoding that is represented by the characteristics of 20 amino acids at different positions in the protein sequence. Given a protein **P**, the Position-Specific Iterated BLAST (PSI-BLAST) program (Altschul et al., 1997[1]) is often used to extract the PSSM descriptor. The occurrence frequency of amino acid residues at a certain site is generated after running the PSI-BLAST algorithm. Several previous studies have indicated that using the PSSM descriptor improves performance in a variety of biological classification studies (Arif et al., 2020[2]; Charoenkwan et al., 2020[14]; Zhang et al., 2015[64]).

### Machine learning algorithms

As indicated in Table 1(Tab. 1), there are three commonly utilized machine learning algorithms (NB, RF, and SVM) in this work. SVM was chosen as the algorithm of choice for creating the current PVP predictors among various ML methods. Meanwhile, in computational biology challenges, SCM-based and ensemble-based approaches are common solutions. The core notions of these five approaches are briefly discussed below.

To determine an unknown sample, the NB method uses the Bayes theorem and a set of conditional independence assumptions (Kumar et al., 2015[33]). NB is known as a probabilistic-based classifier as it computes the predicted class with the maximum probability of investigated features. Given an unknown protein sequence **P**, it is represented with feature vector *F* = (*f*_1_,*f*_2_,…,*f*_n_). Subsequently, its class is predicted by finding out the class *C* that can maximize the likelihood *P*(*F*|*C*) = *P*(*f*_1_,*f*_2_,…,*f*_n_) where *C* = {0,1} that is 1 and 0 represent PVP and non-PVP classes, respectively (Altschul et al., 1997[1]; Kawashima and Kanehisa, 2000[32]; Truong et al., 2015[57]; Wei et al., 2020[60]). As can be seen in Table 3(Tab. 3), NB algorithm was employed in Feng et al.'s method (Feng et al., 2013[27]) and PhagePred (Pan et al., 2018[48]).

SVM is well-known as one of the most effective machine learning algorithms for dealing with binary classification problems, and it has been effectively applied in a variety of domains (Dao et al., 2019[21]; Feng et al., 2019[26]; Lai et al., 2019[35]; Su et al., 2018[54]; Xu et al., 2019[62]; Zhang et al., 2020[65]; Zhu et al., 2019[66]). The Vapnik-Chervonenkis theory of statistical learning was first developed in 1995 (Cortes and Vapnik, 1995[20]; Vapnik, 2013[58]; Vapnik, 1999[59]). It was then expanded to handle the multiclass classification task. Unlike other machine learning algorithms, SVM can reliably generalize the underlying data. In the instance of binary classification, SVM creates a classifier by determining the hyperplane with the greatest distance between two classes (i.e. PVP and non-PVP). In the meanwhile, the kernel function is used to transform the sample space with *p*-dimensional feature vector into the feature space with *n*-dimensional feature vector, where *p < n*. As can be seen in Table 1(Tab. 1), the SVM algorithm is used to construct several of the existing predictors consisting of PVPred (Ding et al., 2014[22]), PVP-SVM (Manavalan et al., 2018[42]), Tan et al.'s method (Tan et al., 2018[55]), Pred-BVP-Unb (Arif et al., 2020[2]), Zhang et al.'s method (Zhang et al., 2015[64]), Meta-iPVP (Charoenkwan et al., 2020[14]) and iPVP-MCV (Han et al., 2021[28]).

Conventional RF-based models were often constructed based on the original RF algorithm as introduced by Breiman ( 2001[5]). These models are constructed by integrating a collection of weak classification and regression tree (CART) classifiers to enhance the predictive performance of CART (Breiman, 2001[5]; Breiman et al., 2017[6]). In RF, the out-of-bag (OOB) approach is used for measuring the invested feature importance. The procedure for the out-of-bag (OOB) approach consists of two main stages as follows: (1) two-thirds of the training sample is employed in the construction of a classifier while the remaining is used for assessing the predictive performance of such classifier and (2) the importance score of each feature is obtained by calculating the decrease in their predictive performance.

The original SCM and ensemble-based SCM methods was firstly introduced by Huang et al. (2012[31]) and Charoenkwan et al. (2013[15]) for predicting and analyzing the protein solubility and protein crystallization, respectively. Recently, Charoenkwan et al. developed an improved version of SCM method known as the flexible scoring card method (FSCM) (Charoenkwan et al., 2021[9]) that provides improved prediction and characterization of anticancer peptides. The procedure for the SCM-based predictor development consists of five main stages (Charoenkwan et al., 2021[7], 2020[10], 2020[11], 2013[15], 2020[17]):(i) preparing training and independent datasets, (ii) calculating initial propensity scores of 20 amino acids and 400 dipeptides, (iii) using the genetic algorithm for obtaining optimal propensity scores of 20 amino acids and 400 dipeptides, (iv) constructing a scoring function based on the optimal propensity scores of 400 dipeptides and (v) predicting the biological functions of unknown protein sequences.

### Feature selection algorithms

Feature selection is an important step in building an effective and robust machine learning model. As indicated in Table 1(Tab. 1), the most popular methodology for selecting the ideal feature sets to create 5 out of 13 existing PVP predictors is a two-step feature selection strategy, which includes PVPred (Ding et al., 2014[22]), PVP-SVM (Manavalan et al., 2018[42]), PhagePred (Pan et al., 2018[48]), Tan et al.'s method (Tan et al., 2018[55]) and Zhang et al.'s method (Zhang et al., 2015[64]). This strategy's procedure is outlined below. The first step is to rate all attributes in order of importance. Analysis of variance (ANOVA) (used in PVPred (Ding et al., 2014[22]), PhagePred (Pan et al., 2018[48]) and Tan et al.'s method (Pan et al., 2018[48])), relief algorithm (used in Zhang et al.'s method (Zhang et al., 2015[64])) and RF algorithm (used in PVP-SVM (Manavalan et al., 2018[42])). The most significant characteristic is the one with the greatest importance scores. The next step is to choose the best feature set. It is also worth noting that all five PVP predictors make use of the incremental feature selection (IFS) for determining the best feature set. Particularly, the IFS strategy's procedure for determining the optimal number of features has two stages: (i) the first feature subset is built using the feature with the highest importance scores and (ii) the second feature subset is built by integrating the first feature subset with features with the second highest importance scores. This method was repeated until all of the researched traits were incorporated, starting with the higher score and ending with the lower score. The feature set with the highest performance is deemed to be the best.

### Performance evaluation and evaluation strategy

To date, the performance of the 13 state-of-the-art PVP predictors has been assessed using three well-known performance evaluation strategies: *K*-fold cross-validation, jackknife validation test/LOOCV, and independent test (Arif et al., 2020[2]; Charoenkwan et al., 2020[11][14]; Ding et al., 2014[22]; Fang and Zhou, 2021[25]; Feng et al., 2013[27]; Han et al., 2021[28]; Manavalan et al., 2018[42]; Pan et al., 2018[48]; Ru et al., 2019[51]; Seguritan et al., 2012[53]; Tang et al., 2016[56]). The performance of these PVP predictors is assessed using the accuracy (ACC), sensitivity (Sn), specificity (Sp), Matthew's correlation coefficient (MCC), and area under receiver operating characteristic (AUC) curves (Arif et al., 2020[2]; Charoenkwan et al., 2020[11][14]; Ding et al., 2014[22]; Fang and Zhou, 2021[25]; Feng et al., 2013[27]; Han et al., 2021[28]; Manavalan et al., 2018[42]; Pan et al., 2018[48]; Ru et al., 2019[51]; Seguritan et al., 2012[53]; Tang et al., 2016[56]). These performance metrics are defined as follows:


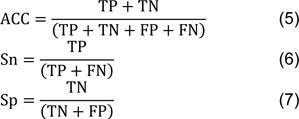


where TP and TN are true positive and true negative, which represent the number of correctly predicted PVPs and non-PVPs. FP is the false positive, which represents the number of non-PVPs predicted as PVPs. FN is the false negative, which represents the number of PVPs predicted as non-PVPs. As for the Sn and Sp metrics, they are used to measure the model's predictive ability in PVPs and non-PVPs, respectively. Moreover, ACC and MCC are used to measure the model's predictive ability for two class problems. 

## Machine Learning-Based PVP Predictors

Table 1(Tab. 1) summarizes 13 state-of-the-art ML-based PVP predictors in terms of ML algorithms, feature selection techniques and performance evaluation strategies. These PVP predictors can be divided into three groups based on the types of machine learning algorithms used. The first group is made up of methods that are based on traditional machine learning algorithms consisting of ANN, NB, SCM, SVM, and RF. An approach based on ensemble learning constitutes the second group. Particularly, the ensemble approach uses two strategies: majority voting and meta-predictor approaches. A deep learning (DL)-based approach makes up the third group.

### Conventional machine learning-based method

Seguritan et al. developed the first PVP predictor based on ANN algorithm (called iVIREONS (Seguritan et al., 2012[53])) for determining viral structure proteins using the primary sequence information namely making use of AAC and PIP descriptors. A year later, Feng et al. developed an NB-based PVP predictor (referred herein as the Feng et al.'s method (Feng et al., 2013[27])) that makes use of AAC and DPC feature descriptors as applied to the *Feng2013* dataset that contained 99 PVPs and 208 non-PVPs. To improve the precision of PVP identification, Feng et al. used the CFS algorithm for determining m informative features from 420 features. The optimal feature set having m informative features consists of V, T, A, H, K, E, R, S, LE, VT, VG, MK, TA, TS, AT, HI, KL, KI, KH, KN, KK, KD, KE, KW, KR, DK, EF, EL, EV, EK, EE, EW, CE, WK, RE, SG, GV and GG. The LOOCV performance (ACC, AUC) was (0.756, 0.758) and (0.792, 0.855), respectively, which made use of a total of 420 features as well as optimal features.

In 2014, Ding et al. introduced an SVM-based PVP predictor named PVPred (Ding et al., 2014[22]). Particularly, PVPred makes use of the GGAP descriptor for distinguishing PVPs from non-PVPs on the *Ding2017* dataset. Specifically, the GGAP's parameter (*g*) was set to be in the range of 0 to 9. Finally, the protein sequence P is represented by a 400-D feature vector for each g. Subsequently, Ding et al. used the ANOVA approach together with the IFS process for determining important features that leads to improvement in the prediction ability of the model. Finally, the optimal feature set was inputted in to SVM algorithm to construct the final model. PVPred achieved a maximum ACC of 0.850 by using the 160 top-ranked GGAP (*g=1*) features. Ding et al. established the first independent dataset containing 11 PVPs and 19 non-PVPs. Particularly, PVPred correctly identified the 9 PVPs and 17 non-PVPs.

In 2018, Manavalan et al. (2018[42]) proposed a novel predictor called PVP-SVM for accurately recognizing PVPs in 2018. PVP-SVM was a PVP predictor based on SVM that worked with AAC, DPC, CTD, ATC, and PCP. To find the best feature set, PVP-SVM used the SVMQA method, which was a systematic feature selection strategy. There were 136 informative features in the best feature set. They were obtained from 8 AAC features, 1 ATC feature, 25 CTD features, 98 DPC features, and 4 PCP features among the 136 relevant features. Cross-validation and independent test (ACC, MCC) results from PVP-SVM were (0.870, 0.695) and (0.798, 0.531), respectively.

PhagePred (Pan et al., 2018[48]) and Tan et al.'s method (Tan et al., 2018[55]) were developed using the GGAP descriptor. Unlike that of PVPred (Ding et al., 2014[22]), PhagePred (Pan et al., 2018[48]) is an NB-based PVP predictor built with the GGAPTree descriptor. The final vector for GGAPTree is represented as a 49220-D feature vector. Particularly, the ANOVA approach together with the IFS process was employed for determining important features as well as for improving the prediction ability of the model. As for Tan et al.'s method, it is an SVM-based PVP predictor that combines the use of ten best feature subsets, which were obtained from the ANOVA and mRMR feature selection methods. The cross-validation and independent test ACC of PhagePred (Pan et al., 2018[48]) and Tan et al.'s method (Tan et al., 2018[55]) provided corresponding values of (0.981, N/A) and (0.880, 0.755), respectively.

Ru et al.'s method (Ru et al., 2019[51]) is an RF-based PVP predictor that is built with AACPCP, AKSNG and Seq-Str. Particularly, this method employs the MRMD approach for determining m informative features from a set of 661 features. This led to identification of the best m number that was found to be 256. The method was found to achieve values of 0.879, 0.963, 0.935 and 0.853 for Sn, Sp, Ac and MCC, respectively, using the 10-fold cross-validation test. In addition, this study also reported that the charge property was the most important physicochemical property for PVP identification.

In 2019, Arif et al. developed an unbiased predictor called the Pred-BVP-Unb (Arif et al., 2020[2]). Particularly, the model was built using the SVM algorithm with the synthetic minority oversampling technique (SMOTE) for solving the imbalance problem on the training dataset (i.e. 99 PVPs and 208 non-PVPs). Moreover, multi-view features containing AAC, SAAC and bi-PSSM were employed to capture the wide array of information of PVPs. An optimal feature set of 86 top-ranked features was selected from amongst an initial set of 502 features for the construction of the final model. Pred-BVP-Unb yielded cross-validation and independent test ACC of 0.925 and 0.831, respectively.

Unlike previous existing PVP predictors, PVPred-SCM (Charoenkwan et al., 2020[11]) is a simple and highly interpretable PVP predictor. Particularly, PVPred-SCM was developed using the SCM method together with DPC descriptors. Furthermore, propensity scores of 400 dipeptides for PVPs were generated and optimized for predicting and characterizing PVPs. Experimental results demonstrated that the performance of PVPred-SCM as evaluated by cross-validation and independent test was found to achieve an ACC of 0.925 and 0.777, respectively, when compared to those of existing SVM-based method (i.e. PVP-SVM (Manavalan et al., 2018[42])) and could outperform a few other PVP predicators such as PVPred (Ding et al., 2014[22]) and Tan et al.'s method (Tan et al., 2018[55]).

### Ensemble-based PVP method

In 2015, Zhang et al. proposed the first stacking-based PVP predictor (called Zhang et al.'s method (Zhang et al., 2015[64])). In their stacking-based PVP predictor, they employed hybrid features consisting of CTD, bi-profile Bayes, PAAC and PSSM. From amongst these four feature descriptors, the bi-profile Bayes descriptor could achieve the best cross-validation performance with ACC of 0.795, MCC of 0.595 and AUC of 0.835. Particularly, bi-profile Bayes afforded the best performance from amongst the four feature spaces with an accuracy of 0.795, an MCC of 0.595 and an AUC of 0.835. In addition, the Relief method was used to construct and rank the 4 feature types. As a result, the optimal feature subsets for CTD, bi-profile Bayes, PseAAC and PSSM consisted of top 79, 55, 32 and 50 features, respectively. The four RF models trained on four optimal feature subsets were integrated and used in the development of the final model using the LR algorithm. On the independent dataset, Zhang et al.'s method provided Sn of 0.853, Sp of 0.815, ACC of 0.831 and MCC of 0.662.

In 2020, our group had developed a novel meta-predictor called the Meta-iPVP (Charoenkwan et al., 2020[11]). Unlike that of Zhang et al.'s method (Zhang et al., 2015[64]), Meta-iPVP combined four different ML algorithms (ANN, NB, RF and SVM) and seven different feature descriptors (AAC, APAAC, DPC, CTDC, CTDD, CTDT and PAAC) for generating 28 baseline models. These baseline models were used to generate 28 PFs. To improve the representation ability of PFs, the GA-SAR algorithm was used to determine the best m number out of 28 PFs. Finally, the 16 selected PFs were used as inputs for training the final meta-predictor using the SVM algorithm. Cross-validation and independent test results (ACC, MCC) of Meta-iPVP were (0.846, 0.698) and (0.817, 0.642), respectively.

Most recently, another ensemble-based PVP predictor (named iPVP-MCV) was proposed by Han et al. (2021[28]). Particularly, three PSSM-based descriptors were found to perform well as compared to that of the Seq-AAC descriptor in terms of four out of five metrics (i.e. ACC, SN, SP, and MCC). In the base layer, three SVM-based models were generated using three different feature encodings (i.e. PSSM-AAC, DP-PSSM and PSSM-composition). In the meta layer, iPVP-MCV integrated predicted classes as derived from these baseline models via the use of the majority voting strategy. The iPVP-MCV approach was applied on the *Manavalan2018* dataset and gave the following results ACC, Sn, Sp and MCC of 0.840, 0.667, 0.922 and 0.621, respectively, as evaluated by independent test. In the meanwhile, when applied on the *Charoenkwan2020_2.0* dataset, iPVP-MCV gave rise to ACC, Sn, Sp and MCC of 0.833, 0.889, 0.778 and 0.671, respectively, as evaluated by independent test.

### Deep learning-based PVP method

To the best of our knowledge, there is one PVP predictor in existence that was developed using the DL algorithm and this is VirionFinder (Fang and Zhou, 2021[25]). Particularly, Fang and Zhou employed the CNN algorithm for model building using one-hot representation and 20 PCPs. Interestingly, this method was effective in identifying both complete and partial PVP from the virome data. Their comparative results with related PVP predictors (i.e. PVPred (Ding et al., 2014[22]), PVP-SVM (Manavalan et al., 2018[42]), PVPred-SCM (Charoenkwan et al., 2020[11]) and Meta-iPVP (Charoenkwan et al., 2020[14])) using their datasets showed that the Sn of VirionFinder was higher than those of the compared PVP predictors on both the complete and partial datasets.

## Performance Comparison and Analysis

**Figure 1:**
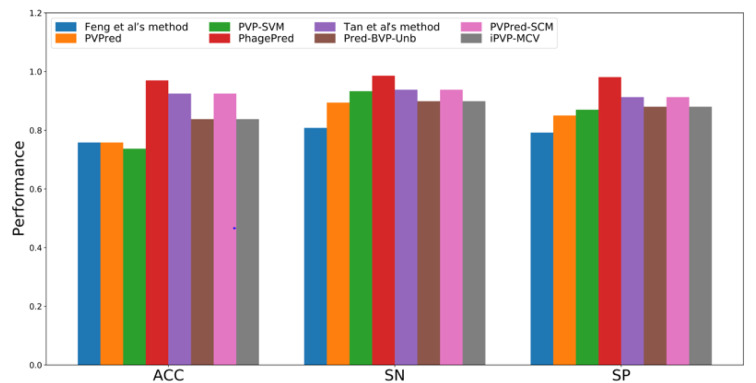
Performance evaluation on the *Feng2013* dataset as deduced from 10-fold cross validation test

**Figure 2:**
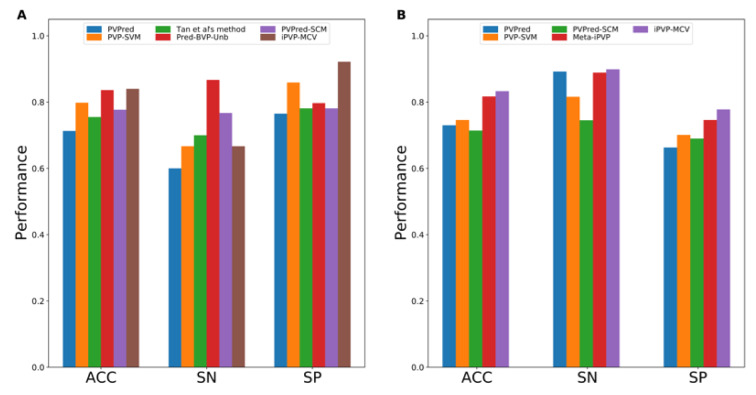
Performance evaluation on Manavalan2018 (A) and Charoenkwan2020_2.0 (B) datasets as deduce from independent test

As can be seen from Table 3(Tab. 3), almost all of the existing PVP predictors were developed and optimized using the three well-known benchmark datasets (i.e. *Feng2013* (Feng et al., 2013[27]), *Manavalan2018* (Manavalan et al., 2018[42]) and *Charoenkwan2020_2.0* (Charoenkwan et al., 2020[11])), with only few exceptions (i.e. iVIREONS (Seguritan et al., 2012[53]), Ru et al.'s method (Ru et al., 2019[51]) and VirionFinder (Fang and Zhou, 2021[25])). Herein, we conducted a comparative analysis of these existing PVP predictors. Cross-validation and independent test results are summarized in Tables 4(Tab. 4) and 5(Tab. 5), respectively. Several of these existing PVP predictors including Feng et al.'s method (Feng et al., 2013[27]), PVPred (Ding et al., 2014[22]), PVP-SVM (Manavalan et al., 2018[42]), PhagePred (Pan et al., 2018[48]), Tan et al.'s method (Tan et al., 2018[55]), Pred-BVP-Unb (Arif et al., 2020[2]), PVPred-SCM (Charoenkwan et al., 2020[11]) and iPVP-MCV (Han et al., 2021[28]), were developed and evaluated using the benchmark dataset through the 10-fold cross-validation test. As can be seen from Table 4(Tab. 4) (References in Table 4: Arif et al., 2020[2]; Charoenkwan et al., 2020[11]; Han et al., 2021[28]) and Figure 1(Fig. 1), PhagePred achieved the highest ACC and MCC of 0.970 and 0.963 while PVPred-SCM (0.938, 0.866) and Pred-BVP-Unb (0.925, 0.850) performed well with the second and third highest ACC and MCC, respectively. For the *Manavalan2018* dataset, six out of eleven existing PVP predictors were built using this dataset (i.e. PVPred (Ding et al., 2014[22]), PVP-SVM (Manavalan et al., 2018[42]), Tan et al.'s method (Tan et al., 2018[55]), Pred-BVP-Un (Arif et al., 2020[2]), PVPred-SCM (Charoenkwan et al., 2020[11]), iPVP-MCV (Han et al., 2021[28])). Table 5(Tab. 5) (References in Table 5: Arif et al., 2020[2]; Charoenkwan et al., 2020[11][14]; Han et al., 2021[28]) and Figure 2A(Fig. 2) show that iPVP-MCV and Pred-BVP-Unb could achieve the best independent test results as evaluated by ACC (0.836-0.840) and MCC (0.621-0.660). PVP-SVM could perform well with the second highest ACC and MCC of 0.798 and 0.531, respectively. In the meanwhile, five out of eleven existing PVP predictors were evaluated by the *Charoenkwan2020_2.0* dataset and this included PVPred (Ding et al., 2014[22]), PVP-SVM (Manavalan et al., 2018[42]), PVPred-SCM (Charoenkwan et al., 2020[11]), Meta-iPVP (Charoenkwan et al., 2020[11]) and iPVP-MCV (Han et al., 2021[28]). Meta-iPVP and iPVP-MCV could achieve the best independent test results in terms of ACC (0.817-0.833) and MCC (0.642-0.671) (Figure 2B(Fig. 2)).

From Tables 4(Tab. 4) and 5(Tab. 5), several observations can be made. Firstly, iPVP-MCV and Pred-BVP-Unb were found to provide the best independent test results as evaluated on both Manavalan2018 and *Charoenkwan2020_2.0* datasets. However, no web server was provided from these two PVP predictors. Hence, their utility and usage is quite limited. Second, although, PhagePred achieved the best cross-validation results on the *Feng2013* dataset, this predictor did not provide the independent test. It could be stated that PhagePred might not be a suitable tool for identifying candidate PVPs from large-scale proteins. Third, PVP-SVM and Meta-iPVP yielded relatively predictive performance to iPVP-MCV and Pred-BVP-Unb on both Manavalan2018 and Charoenkwan2020_2.0, respectively. In the meanwhile, these two PVP predictors were deployed as a user-friendly web server (http://www.thegleelab.org/PVP-SVM/PVP-SVM.html and http://camt.pythonanywhere.com/Meta-iPVP). Altogether, these comparative results indicated that PVP-SVM and Meta-iPVP could outperform iPVP-MCV and Pred-BVP-Unb as well as other existing PVP predictors in terms of their prediction results and community utility.

## Characterization of Phage Virion Proteins

From amongst the eleven current PVP predictors, PVPred-SCM as introduced by Charoenkwan et al. ( 2020[11]) represents a simple and easily interpretable approach (Charoenkwan et al., 2021[7], 2020[10], 2020[11], 2013[15], 2020[17]). Particularly, the PVPred-SCM model was built using 99 PVPs and 208 non-PVPs as derived from *Feng2013* dataset and their PVP scores were calculated using the scoring function S(P). Results from Charoenkwan et al. (2020[11]) indicated that four of ten proteins having the highest PVP scores were capsid protein (capsid protein G8P, capsid protein G8P, G VIII capsid protein precursor, and major coat protein). In addition, the SCM-derived propensity score of 20 amino acids and 400 dipeptides for PVPs were determined for analyzing the biochemical and biophysical properties of PVPs (Charoenkwan et al., 2020[11]). Charoenkwan et al. ( 2020[11]) reported that Ala, Thr, Val, Gly and Ser were the five top-ranked amino acids with the highest propensity scores of 529.50, 511.43, 506.88, 506.68 and 504.63, respectively, while Leu, Arg, His, Glu, and Lys were found to be amongst the five top-ranked amino acids with the lowest propensity scores. This finding was consistent with results reported by Ding et al. (2014[22]). Particularly, in their study it was found that from amongst the GGAP (*g=1*) features, Ala, Gly, Pro, Ser, Thr were beneficial for PVPs while Glu, Lys, Leu and Arg were beneficial for non-PVPs. Moreover, informative PCPs from the AA index were also determined for analyzing important characteristics of PVPs. Results showed that alpha-helix propensity (KOEP990101) and hydrophobicity index (WOLR790101) were crucial properties of PVPs. Particularly, KOEP990101 and WOLR790101 properties exhibited high positive correlations of 0.502 and 0.484, respectively, while the side-chain of amino acids exhibited high negative correlation of -0.516. Two of five top-ranked amino acids having the highest propensity scores (i.e. Gly and Thr) were found to have high alpha-helix propensity with ranks of propensity scorers (PS, alpha-helix) of (4, 1) and (2, 3), respectively. Moreover, the helix propensity of amino acids was mentioned to be amongst the important contributors of protein stability as rationalized by strong H-bond and Van der Waals interactions (Pace and Scholtz, 1998[47]). Moreover, important amino acids (i.e. Ala, Val and Gly) were found from amongst the ten top-ranked highest propensity scorers and hydrophobicity index as (1, 5), (3, 4) and (4, 1), respectively. Several studies have highlighted the importance of hydrophobic side chain amino acids in the stability of procapsids and phage virions. Gly and Ala were discovered in 50-residues, which were necessary for the inclusion of the M13 filamentous bacteriophage coat (Roth et al., 2002[50]). Furthermore, Ala substitution at Glu52, Glu59, and Glu72 in the coat protein E-loop enhanced the stability of procapsids and virions of bacteriophages P22 (Asija and Teschke, 2019[3]). According to these results, PVPs favored amino acids with high alpha-helix propensity and hydrophobic side index. It was also found that the side-chain property of amino acids were negatively correlated with PVPs. Charoenkwan et al. (2020[11]) reported that Ala, Thr, Val, Gly and Ser had propensity scorer (PS, side-chain) ranks as follows: (1,19), (2,15), (3,16), (4,20) and (5,18), respectively. The role of each amino acid in this protein was studied by performing random mutations at the N-terminal region of fusion phage protein containing the β-galactosidase-binding peptide. The findings revealed that short amino acids play a crucial role in providing a high binding affinity for the principal coat protein's domain C (Kuzmicheva et al., 2009[34]). PVPs favored short amino acids because they had a low radius of octapeptide composing domain C, which can build alpha helix areas and have low steric hindrances, resulting in a low conformation number.

## Prospective Strategies for Improving the Prediction Performance of PVPs

To date, there are 13 ML-based PVP predictors which have been proposed and developed for predicting and analyzing PVPs using primary sequence information only. From amongst these predictors, there were only four PVP predictors that were deployed as a web server (i.e. PVPred (Ding et al., 2014[22]), PVP-SVM (Manavalan et al., 2018[42]), PVPred-SCM (Charoenkwan et al., 2020[11]) and Meta-iPVP (Charoenkwan et al., 2020[11])). In the meanwhile, only one PVP predictor (i.e. PVPred-SCM (Charoenkwan et al., 2020[11])) could provide mechanistic understanding on the underlying properties governing PVPs and this was made possible via the use of SCM-derived propensity scores of 20 amino acids and 400 dipeptides. Although current PVP predictors could achieve an accurate and stable performance, there are still under explored aspects that can help to improve the identification of PVPs. Firstly, sufficient size of training datasets are often needed to enhance the predictive performance of the model. Although several research groups have made efforts in constructing up-to-date PVP datasets (Charoenkwan et al., 2020[11]; Ding et al., 2014[22]; Manavalan et al., 2018[42]), the relative size of PVPs is not of satisfactory level. Secondly, a number of sequence-based feature descriptors were used for the development of current PVP predictors. However, these feature descriptors had certain shortcomings (Charoenkwan et al., 2021[12]). Thus, there is the need to employ a built-in feature extractor for encoding PVPs. Several previous studies have demonstrated a natural language processing (NLP)-based technique (such as TF-IDF, Pep2Vec and FastText) that is known to be an effective built-in feature extractor technique, which is able to achieve an outstanding level of performance when compared to well-known sequence-based feature encodings (Charoenkwan et al., 2021[12]; Le et al., 2019[37]; Li et al., 2020[39]; Nguyen et al., 2020[45]). Thirdly, it is highly desirable to utilize a feature representation learning (FRL) algorithm that can combine variant sequence-based feature descriptors together with ML algorithms in providing class information or probabilistic information. The original FRL algorithm was proposed by Wei et al. (2018[61]), which was developed using several single feature-based SVM-based models. Recently, Charoenkwan et al. (2021[8]), Basith et al. (2021[4]) and Hasan et al. (2021[29]) extended the FRL algorithm of Wei et al. ( 2018[61]) by integrating various ML algorithms such as ANN, KNN, NB and SVM. Fourthly, DL techniques have been demonstrated to be powerful ML techniques that could achieve good level of performance for various biological and chemical classification problems. Although VirionFinder (Fang and Zhou, 2021[25]) is a DL-based PVP predictor that was developed using the CNN algorithm, however its DL structure is quite simple. Thus, there is the need to develop a more comprehensive DL structure.

## Conclusions

This study surveyed and evaluated all currently available ML-based predictors for PVP prediction and characterization. We examined, assessed, and ranked all known PVP predictors in terms of their training/independent datasets, feature encoding algorithms, feature selection methods, core algorithms, performance evaluation metrics/strategies, and website. We used three benchmark datasets in a comparison analysis to find the best PVP predictor. PVP-SVM and Meta-iPVP were found to exceed other existing PVP predictors in terms of effectiveness, according to a comparison investigation. PVP-SVM and Meta-iPVP were found to exceed other existing PVP predictors in terms of effectiveness, acceptability and community utility. This research also provides useful information and future directions for the design and development of new and more sophisticated computational approaches for identifying and characterization of PVPs. We hope that this review will be useful to researchers in identifying the best PVP predictor for their needs as well as assisting in the rapid identification of phage virion proteins.

## Declaration

### Ethical statement

This review paper does not include animal or human experiments.

### Conflict of interest

The authors declare no conflict of interest.

### Author contributions' statement

W.S. - conceptualization, project administration, supervision, investigation, manuscript preparation and revision

M.K. - data analysis; data interpretation, manuscript preparation

S.K. and P.C. - manuscript preparation

C.N. - manuscript revision.

All authors reviewed and approved the manuscript.

### Acknowledgments

This work was fully supported by College of Arts, Media and Technology, Chiang Mai University and partially supported by Chiang Mai University and Mahidol University.

## Figures and Tables

**Table 1:**
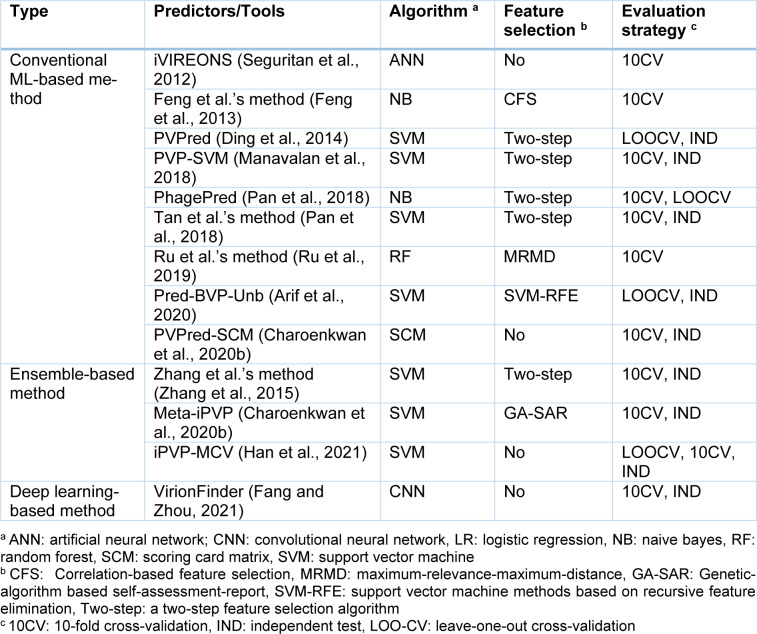
A comprehensive list of current PVP predictors reviewed in this study

**Table 2:**
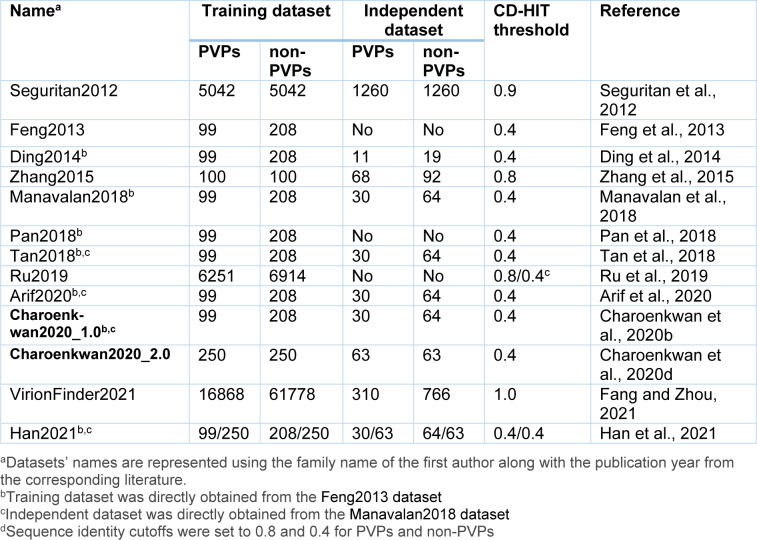
A summary of training and independent test datasets used in PVP predictors

**Table 3:**
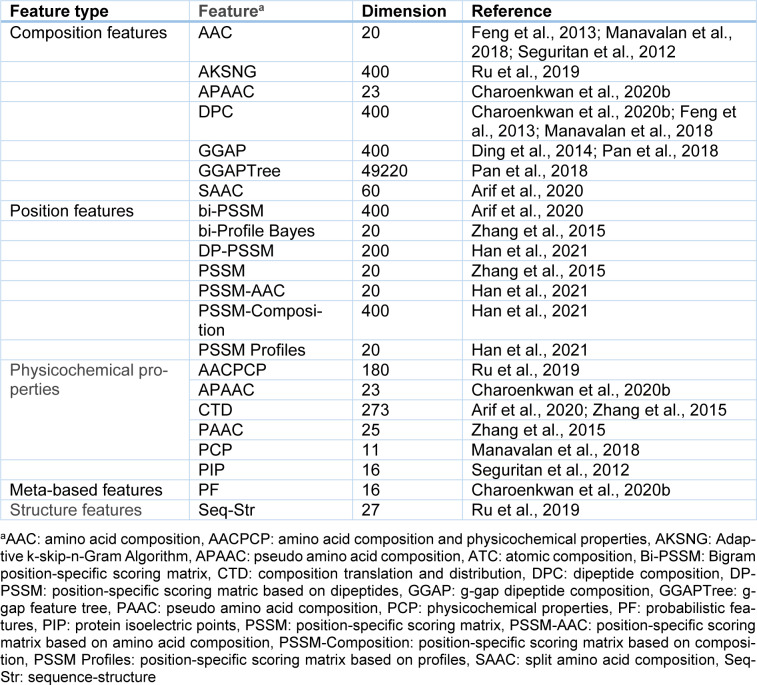
Different types of features employed for developing the PVP predictors

**Table 4:**
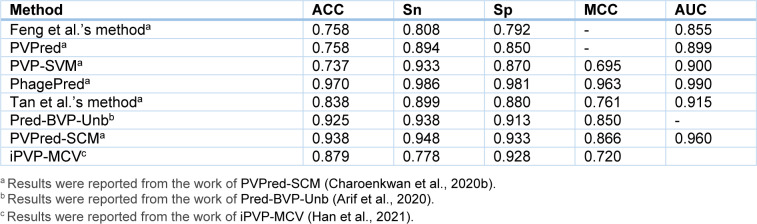
Cross-validation results for different PVP predictors evaluated on the *Feng2013* dataset

**Table 5:**
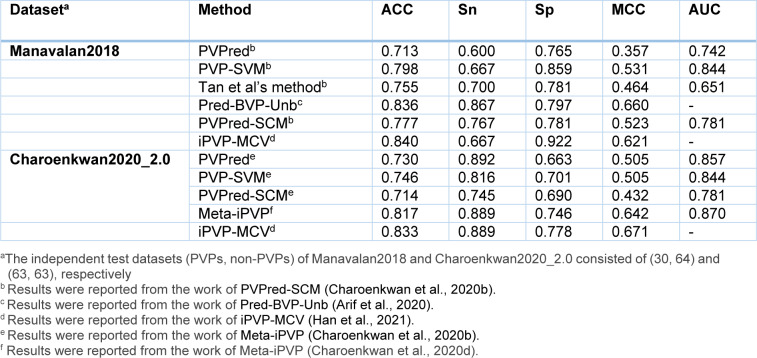
Independent test results from different PVP predictors as evaluated on *Manavalan2018* and *Charoenkwan2020_2.0 datasets*
